# TSLP signaling pathway map: a platform for analysis of TSLP-mediated signaling

**DOI:** 10.1093/database/bau007

**Published:** 2014-02-25

**Authors:** Jun Zhong, Jyoti Sharma, Rajesh Raju, Shyam Mohan Palapetta, T. S. Keshava Prasad, Tai-Chung Huang, Akinori Yoda, Jeffrey W. Tyner, Diederik van Bodegom, David M. Weinstock, Steven F. Ziegler, Akhilesh Pandey

**Affiliations:** ^1^McKusick-Nathans Institute of Genetic Medicine, Johns Hopkins University School of Medicine, 733 N. Broadway, Baltimore, MD 21205, USA, ^2^Department of Biological Chemistry, Johns Hopkins University School of Medicine, 733 N. Broadway, Maryland, 21205, USA, ^3^Department of Oncology, Johns Hopkins University School of Medicine, 733 N. Broadway, Maryland, 21205, USA, ^4^Department of Pathology, Johns Hopkins University School of Medicine, 733 N. Broadway, Maryland, 21205, USA, ^5^Institute of Bioinformatics, International Technology Park, Bangalore 560066, India, ^6^Manipal University, Madhav Nagar, Manipal 576104, India, ^7^Centre of Excellence in Bioinformatics, School of Life Sciences, Pondicherry University, Puducherry 605014, India, ^8^Department of Medical Oncology, Dana-Farber Cancer Institute, Harvard Medical School, 44 Binney Street, Boston, MA 02115, USA, ^9^Division of Hematology and Medical Oncology, Knight Cancer Institute, Oregon Health and Science University, 3181 SW Sam Jackson Park Road, Mailcode L592, Portland, OR 97239, USA and ^10^Immunology Program, Benaroya Research Institute at Virginia Mason, 1201 9th Avenue S&C, Seattle, WA 98101, USA

## Abstract

Thymic stromal lymphopoietin (TSLP) is a four-helix bundle cytokine that plays a critical role in the regulation of immune responses and in the differentiation of hematopoietic cells. TSLP signals through a heterodimeric receptor complex consisting of an interleukin-7 receptor α chain and a unique TSLP receptor (TSLPR) [also known as cytokine receptor-like factor 2 (CRLF2)]. Cellular targets of TSLP include dendritic cells, B cells, mast cells, regulatory T (Treg) cells and CD4+ and CD8+ T cells. The TSLP/TSLPR axis can activate multiple signaling transduction pathways including the JAK/STAT pathway and the PI-3 kinase pathway. Aberrant TSLP/TSLPR signaling has been associated with a variety of human diseases including asthma, atopic dermatitis, nasal polyposis, inflammatory bowel disease, eosinophilic eosophagitis and, most recently, acute lymphoblastic leukemia. A centralized resource of the TSLP signaling pathway cataloging signaling events is not yet available. In this study, we present a literature-annotated resource of reactions in the TSLP signaling pathway. This pathway map is publicly available through NetPath (http://www.netpath.org/), an open access signal transduction pathway resource developed previously by our group. This map includes 236 molecules and 252 reactions that are involved in TSLP/TSLPR signaling pathway. We expect that the TSLP signaling pathway map will provide a rich resource to study the biology of this important cytokine as well as to identify novel therapeutic targets for diseases associated with dysregulated TSLP/TSLPR signaling.

**Database URL: http://www.netpath.org/pathways?path_id=NetPath_24**

## Introduction

Thymic stromal lymphopoietin (TSLP) is a type I cytokine that belongs to the interleukin-2 cytokine family. TSLP signaling requires a heterodimeric receptor complex composed of interleukin-7 receptor α chain (Gene Symbol, *IL7R*) and the TSLP receptor subunit (TSLPR; Gene Symbol, *CRLF2*), which is similar to the common γ chain (1, 2). TSLP was first identified from the conditioned medium of the murine thymic stromal cell line, Z210R.1, as a growth factor that supported B lymphopoiesis *in vitro* (3, 4). TSLP is widely expressed *in vivo* including epithelial cells of the lung, skin and gut, Hassall's corpuscles in the thymic medulla, mucosa-associated lymphoid tissues and tonsils. TSLP is also expressed by primary skin keratinocytes, smooth muscle cells and lung fibroblasts (5–7). At the organ level, it has been shown that heart, liver, spleen and prostate have higher expression levels of TSLP compared with lung, skeletal muscle, kidney, spleen, ovary, small intestine and colon (8).

TSLP can execute its biological functions through its action on many different types of cells. TSLP can activate CD4+ T cells (9) and CD8+ T cells in mice (10) and induces B-cell proliferation and differentiation in humans (11). It also enhances maturation and proliferation of dendritic cells and naive T-cells, respectively. It has also been shown to induce the release of T-cell attracting chemokines from monocytes. In combination with interleukin-1 and tumor necrosis factor, TSLP can stimulate the production of Th2 cytokines by human mast cells (12).

On binding its receptor complex, TSLP can activate multiple signal transduction pathways. Previously, studies have shown that stimulation of IL7R/TSLPR complex by TSLP induces the phosphorylation and activation of Janus kinases (JAKs). Activated JAKs, in turn, regulate the activity of signal transducers and activators of transcription (STAT) factors, which include STAT1, STAT3, STAT4, STAT5a, STAT5b and STAT6 (13–15). In addition, several other proteins such as AKT1, ERK1/2, JNKs, ribosomal protein S6 kinase and 4E-BP1 have also been shown to be activated on TSLP stimulation (10, 13, 16, 17). Recently, our group carried out SILAC-based quantitative phosphoproteomic analysis to identify molecules that are differentially phosphorylated on stimulation with TSLP. Our results revealed that TSLP can regulate phosphorylation of 226 proteins including several members of the SRC and TEC family of kinases and protein phosphatases such as PTPN6 (Protein tyrosine phosphatase non-receptor type 6, also called SHP-1) and PTPN11 (Protein tyrosine phosphatase non-receptor type 11, also called SHP-2) (18).

TSLP and TSLPR have been implicated in a number of pathological conditions. TSLP expression was found to be increased in asthmatic airways and its overexpression correlated with severity of disease (19, 20). TSLP was reported to be upregulated in keratinocytes of atopic dermatitis patients (21). Overexpression of TSLP has been shown to be associated with the development of nasal polyps (22). TSLP has also been implicated in the regulation of intestinal immunity and inflammation in a mouse model of inflammatory bowel disease (23). Rothenberg and colleagues have reported that TSLP is the most likely candidate gene responsible for pathogenesis of eosinophilic esophagitis (24). Several studies also show the involvement of the TSLPR gene in leukemia (25–30). Yoda and colleagues have shown that high expression of TSLPR results from translocation of the immunoglobin heavy chain locus or interstitial genomic deletions in B-cell acute lymphoblastic leukemia (31). An activating mutation in TSLPR, Phe232Cys, has also been found to be associated with this leukemia (31, 32).

Despite the importance of the TSLP signaling pathway, the reactions specific to TSLP signaling reported to date are not available in any public pathway resource. Kyoto Encyclopedia of Genes and Genomes (KEGG) database (33) contains pathways through several cytokine receptors including receptors for TSLP; however, KEGG represents generic signaling modules that are restricted to JAK-STAT, RAS-RAF-MAPK and PI3K-AKT and does not have a pathway that is specific to TSLP signaling. Even these generic signaling modules in KEGG contain only 25 molecules and 30 reactions, without any link to corresponding research articles. Our catalog contains 236 molecules and 252 reactions stimulated by TSLP through literature search and manual annotation, which is made available through NetPath (34) (http://www.netpath.org/pathways?path_id=NetPath_24). NetPath is an open access signaling pathway resource that provides a list of all of the reactions curated from the literature to present a comprehensive and global view of signaling pathways. We have also generated a high-confidence list and visualization of TSLP pathway that is available through NetSlim (35) (http://www.netpath.org/netslim/NetSlim_24). NetSlim uses predefined criteria to show only high-confidence reactions as a means to generate a ‘slim’ version of the pathway, which is less complex and more easily visualized.

## Annotation of TSLP signaling pathway reactions

An extensive search of published literature was carried out in PubMed to annotate reactions involved in the TSLP signaling pathway. The query terms used are listed in the Appendix. Research articles were screened for information about reactions such as protein–protein interactions (PPIs), posttranslational modifications (PTMs), translocation and activation/inhibition of proteins, which occur on stimulation with TSLP. We also looked for genes that are differentially regulated on stimulation by TSLP in humans. The reactions stimulated by TSLP as compared with an unstimulated state (reactions induced on co-stimulation with TSLP and any other stimuli were excluded) in human or other mammalian systems were included. We used PathBuilder (36), a pathway annotation tool developed by our group, to annotate reactions in TSLP signaling. Reactions were captured under the following categories:

## Protein–protein interactions

Proteins interact with other proteins and nonproteins and mediate transduction of extracellular signals into the interior of the cell. Physical association events between proteins that occur on TSLP stimulation were captured under this category. These were classified as either ‘binary’ or ‘complex’ (multimeric) associations. The PPIs induced by TSLP were considered ‘binary’ if homomeric/heteromeric interactions are reported between two proteins or if only two proteins are studied/identified in coprecipitation assays. Multimeric association of proteins consisting more than two components was considered as ‘complex’ interactions. For example, biochemical interaction between TSLP and IL-7 receptor alpha subunit and TSLPR has been reported by several groups (2). More recently, Zhong *et al.* (18) have shown that TSLP can induce the interaction between SHP-2 and GAB2 (GRB2-associated binding protein 2). Such PPI events have been annotated with a link to the corresponding references.

## Enzyme–substrate reactions

PTMs are dynamic covalent modifications on proteins. PTMs are important for regulation and integration of cellular signaling events. PTMs regulate PPIs, activity of enzymes and the cellular localization of proteins. Under this category, we have annotated enzyme-catalyzed reactions that have been reported to be stimulated by TSLP. Like other cytokines, TSLP also uses the JAK-STAT signaling module to transmit signals in cells by inducing phosphorylation of some members in the module, including JAK1, JAK2, STAT1, STAT3, STAT4, STAT5A, STAT5B and STAT6 (13–15, 37, 38). Apart from the JAK-STAT signaling module, TSLP also induces activation of the PI3K-AKT-mTORC1 (10, 13, 16, 39–41), SRC/TEC (42), ERK (13, 17, 40, 41, 43), NFκB (13), JNK1/2 (17) and p38MAPK (17) signaling modules in diverse cell types. More recently, a quantitative phosphoproteomics study carried out by our group revealed TSLP-induced changes in phosphorylation status of 226 molecules (18). Among them, catalysis events for which the immediate upstream enzyme is proven in the study were classified as ‘direct’ reactions. Reactions for which the upstream enzyme is not proven in the study were classified as ‘induced’ catalysis events. The site and residue of the PTM was also captured when available.

## Protein translocation events

Movement of proteins across subcellular compartments is critical for the function of the proteins as well as for the transfer of information inside the cell. These translocation events may depend on a variety of factors, which include PTMs or association/dissociation with other molecules. For example, TSLP signaling in dendritic cells also leads to activation and nuclear transport of NFκB1, NFκB2, RELA and RELB proteins to induce the production of OX40L (13). The proteins that undergo translocation on the stimulation of TSLP were listed in this category. The source and target cellular compartments of the translocated proteins were also documented.

## Activation/inhibition reactions

Activation and inhibition of proteins, at the human interface, are identified by activity assays. This category lists proteins whose activity is found to be altered on TSLP treatment. When the activity of proteins has been shown to be changed by TSLP stimulation through activation/inhibition assays, such as using small kinase inhibitors or through the use of intrinsic or extrinsic substrates, these proteins were included under the activation/inhibition category. For example, the involvement of SRC and TEC family of kinases in the TSLP induced proliferation of B cells has been demonstrated through pharmacological inhibitors (42, 44).

## Gene regulation events

Most stimuli bring about their phenotypic effects by altering the gene expression in the target cell. Under this category, we annotated genes whose expression levels are reported to change in response to TSLP treatment. Gene expression changes at the mRNA level in human cells/tissues proved using various techniques including DNA microarray and northern blot were captured. In addition, transcriptional regulators of these genes that are differentially regulated were also annotated, if proven in the study. For example, induction of STAT3 by TSLP regulates the expression of *NME1* and *MYC* in trophoblasts and of *IL6*, *IL8* and *CCL11* in smooth muscle cells (17, 45). In T lymphocytes, TSLP-induced STAT5 regulates the expression of *CISH* and *IL2RA* (45, 46).

In all the above-mentioned categories, gene symbols and NCBI Gene identifiers were used to represent the molecules involved in reactions. We have also documented additional information for reactions such as (i) species of the protein(s) and cell line/type used to prove the reaction; (ii) the cellular compartment where the reaction takes place; (iii) the experiment type, i.e. whether the reaction was carried out *in vivo* or *in vitro*; and (iv) the PubMed identifier of the study where the reaction was described. We have also given a brief description for each of the reactions under the ‘Comments’ section.

## Summary of TSLP pathway annotation

Our literature survey and annotation of articles resulted in the identification of 236 proteins that are involved in mediating the effects of TSLP. These molecules were found to be involved in 9 PPIs, 232 catalysis events, 7 translocation events and 4 activation events. The biomedical significance and the relatively sparse information available for this important pathway in the literature, underscore the need for additional systematic studies required to identify the effectors of this pathway and their biological roles. Most enzyme-substrate reactions that we cataloged were ‘indirect’ and involve phosphorylation or dephosphorylation events. The site and residue information of PTMs was annotated for 155 proteins. We also cataloged 91 genes, which were transcriptionally regulated on TSLP stimulation in primary human cells or cell lines. As mentioned above, these reactions are linked to the corresponding published literature where they were described. The pathway reactions were reviewed both internally and by an external pathway authority to ensure availability of high-quality data to the biomedical research community.

## Data formats, availability and visualization

The TSLP pathway webpage in the NetPath includes a description of the pathway as well as statistics of the number of molecules and reactions. The pathway reactions are encoded in various community standard data exchange formats such as Proteomics Standards Initiative for Molecular Interaction (PSI-MI version 2.5) (47), Biological PAthway eXchange (BioPAX level 3) (48) and Systems Biology Markup Language (SBML version 2.1) (49). Users can download these files from NetPath and import them in various freely available software such as Cytoscape (50), VISIBIOweb (51) and ChiBE (52) to visualize the pathway reactions. The data are also available in tab delimited and Microsoft Excel formats. The TSLP pathway in NetPath can also be accessed through WikiPathways (53), another open collaborative platform dedicated to the curation of biological pathways.

## The TSLP signaling pathway map

**Figure 1.**
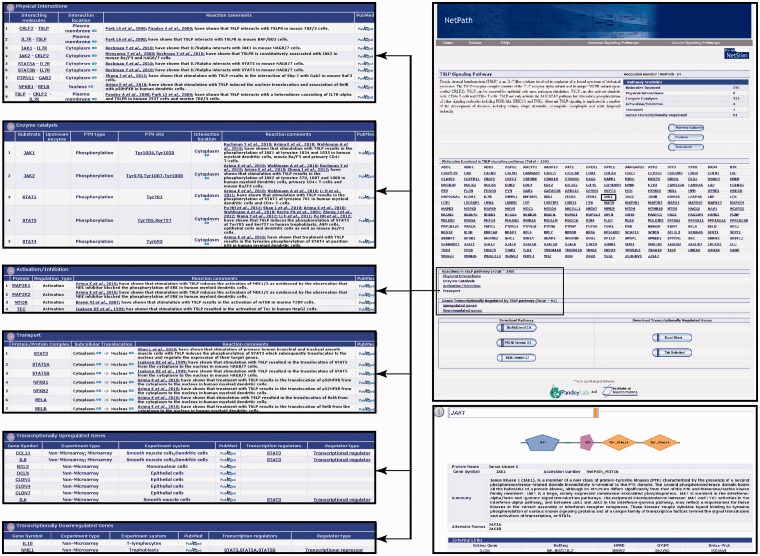
An overview of the TSLP pathway page in NetPath. The TSLP pathway page in NetPath provides statistics pertaining to the number of molecules curated, links to TSLP pathway reactions and a list of genes that are differentially regulated by TSLP. Each molecule in the pathway page is linked to the corresponding NetPath molecule page, which is further linked to Entrez Gene, HPRD, OMIM and Swiss-Prot identifiers. The reaction page of the TSLP pathway contains a list of each type of reaction such as physical interactions, enzyme catalysis or transport with a brief description of the reactions including their PTM dependence or interacting regions/domains/motifs whenever available in literature. The list of curators and reviewers are provided in the TSLP pathway page with the details of the pathway authority. A comments tab is also provided.

Even though pathway reactions can be visualized and analyzed using the above-mentioned software, these do not give directionality to the reactions involved. Also, in many cases the relationship among molecules is not clear or linked to canonical signaling pathways (Figure 1). This makes it difficult for the user to interpret the pathway. To circumvent this problem, we have also developed a high-confidence signaling map version of TSLP pathway. The high-confidence reactions were filtered using the criteria described previously by our group (35). The directionality of molecular reactions were devised based on TSLP-induced signaling studies (for instance from studies that use inhibition assays) (17, 40, 54). The pathway map was made using PathVisio, a freely available pathway drawing software (55).

**Figure 2.**
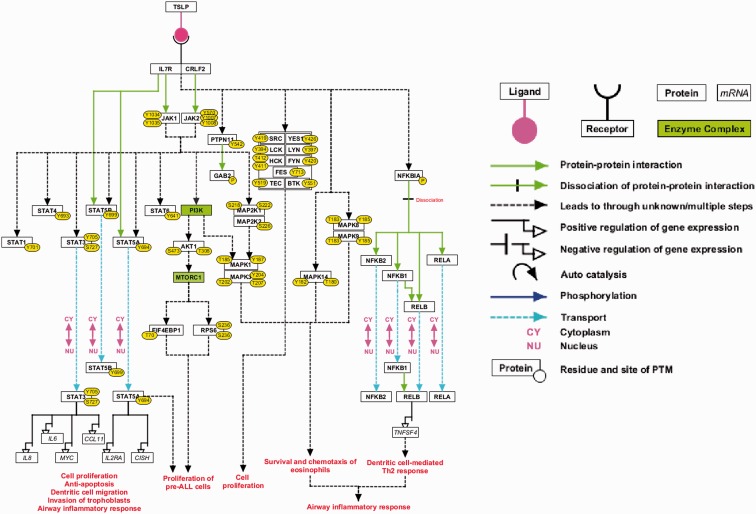
A high-confidence reaction map of the TSLP signaling pathway. The pathway reaction map contains 37 proteins involved in 9 molecular associations, 21 enzyme catalysis reactions and 7 translocation events. Apart from the reactions selected based on NetSlim criteria, we have also included 2 enzyme complexes namely MTORC1 and PI3-kinase complex based on the inhibitor assays that confirmed their role in TSLP signaling and our phosphoproteomics study (the nodes highlighted in green). The edges representing the relationships between nodes are explained in the legend.

This high-confidence NetSlim pathway map contains 37 molecules involved in the TSLP/TSLPR complex signaling system (Figure 2). Two enzyme families—SRC and TEC—along with two enzyme complexes, PI3K and MTORC1, have been studied in TSLP signaling through the use of small molecule inhibitors (5, 13, 16, 44, 56). These have been included in the high-confidence TSLP signaling map. This pathway map is available as part of the NetSlim resource (35) and can be accessed at http://www.netpath.org/netslim. These pathway data are provided in all the exchange formats mentioned above. Apart from these, the pathway map is also provided in .pml, GenMAPP (57), .ng and .df formats.

## Update process

The TSLP signaling pathway will be regularly updated, as additional signaling events are reported in the literature. The query terms in the Appendix will be used to search for newer studies pertaining to the TSLP signaling pathway as they are published. The pathway map will also be annually updated based on NetSlim criteria by inclusion of further molecules and their reactions as more studies report them in the future. We hope that the biomedical research community will actively participate in the continuous update and improvement of the TSLP signaling pathway resource and map provided here.

## Conclusions

Availability of TSLP signaling reactions in a single resource will aid in understanding the role of various molecules in the biology of this pathway. Such data can also be used to analyze high-throughput data from various platforms including DNA/protein microarrays and mass spectrometry-based proteomics experiments. These data have been submitted to the NetPath resource and are made available in diverse community standard data exchange formats so that they can be easily visualized and analyzed. We hope that this resource will help in designing experiments aimed at expanding the existing knowledge of the TSLP signaling in both normal and disease physiology. We encourage active participation of researchers to improve the quality and content of our resource. The scientific community can give their suggestions and comments through NetPath (http://www.netpath.org/comments).

## Funding

National Heart Lung and Blood Institute (HHSN268201000032C to A.P.) and an NIH Roadmap grant ‘Technology Center for Networks and Pathways’ (U54 GM103520 to A.P.). Senior Research fellowship award from Council of Scientific and Industrial Research (CSIR) (to J.S. and S.M.P.).

*Conflict of interest*. None declared.

## Appendix

Thymic stromal lymphopoietin

TSLP

TSLP cytokine

CRLF2

Cytokine receptor-like molecule 2

Cytokine receptor-like factor 2

TSLPR

Thymic stromal lymphopoietin receptor

TSLP signaling pathway
